# Supplementary material for: miR-185-5p Regulates Inflammation and Phagocytosis through CDC42/JNK Pathway in Macrophages
Source: Genes (Basel). 2022 Mar 7;13(3):468. doi: 10.3390/genes13030468 (PMC8955717; doi:10.3390/genes13030468)
Supplement: Supplementary file 1 [file genes-13-00468-s001.zip › genes-1606105-supplementary.pdf]

A

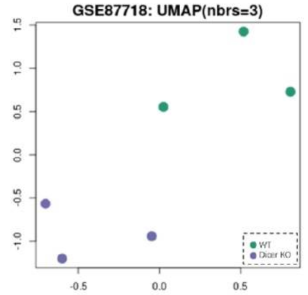

B

Sample	Treatment	Value
GSM4275315	RAW264.7 untreated	2.20038
GSM4275316	RAW264.7 untreated	0.8914
GSM4275317	RAW264.7 untreated	0.710123
GSM4275318	RAW264.7 treated with LPS(100ng/ml) and INFγ(20ng/ml) for 24h	-3.24819
GSM4275319	RAW264.7 treated with LPS(100ng/ml) and INFγ(20ng/ml) for 24h	-3.30166
GSM4275320	RAW264.7 treated with LPS(100ng/ml) and INFγ(20ng/ml) for 24h	-3.31062

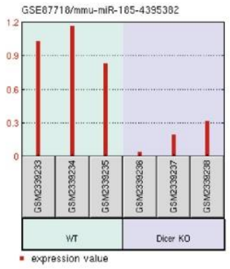

C

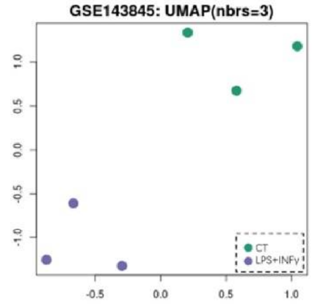

D

Sample	Treatment	Value
GSM2339233	Dicer WT BMDMs	1.0274
GSM2339234	Dicer WT BMDMs	1.16393
GSM2339235	Dicer WT BMDMs	0.836246
GSM2339236	Dicer KO BMDMs	0.044894
GSM2339237	Dicer KO BMDMs	0.198379
GSM2339238	Dicer KO BMDMs	0.318935

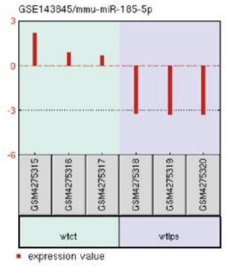

E

Mature sequence mmu-miR-185-5p	
Accession	MIMAT0000214
Previous IDs	mmu-miR-185
Sequence	7 - uggagagaaaggcaguuccuga - 28<br>Get sequence
Deep sequencing	600847 reads, 105 experiments
Evidence	experimental; cloned [1-2], Illumina [3-4]
Database links	RNAcentral: URS00004176D4_10090
Predicted targets	TargetScanVert: mmu-miR-185-5p<br>miRDB: mmu-miR-185-5p<br>microrna.org: mmu-miR-185-5p

F

Conserved Predicted Binding Sites

	Predicted consequential pairing of target region (top) and miRNA (bottom)	Site type	Context++ score
Position 645-652 of Cdc42 3' UTR<br>mmu-miR-185-5p	5' ...UUGAAAGUUGCCUUUUCUCUCCA...<br>|||||| ||||||| 3' AGUCCUUGACGGAA-AGAGAGGU	8mer	-0.52

Supplementary Figure S1. UMAP **(A)** and expression values **(B)** of miR-185-5p in GSE87718. UMAP **(C)** and expression values **(D)** of miR-185-5p in GSE143845. **(E)**The mmu-miR-185 is the previous ID of mmu-miR-185-5p in miRBase. **(F)** Concerved binding sites predicted between miR-185-5p and CDC42 3' UTR by TargetScan.
